# Subtractive genomic analysis for computational identification of putative immunogenic targets against clinical *Enterobacter cloacae* complex

**DOI:** 10.1371/journal.pone.0275749

**Published:** 2022-10-13

**Authors:** Negin Bolourchi, Sepideh Fereshteh, Narjes Noori Goodarzi, Farzad Badmasti

**Affiliations:** 1 Department of Bacteriology, Pasteur Institute of Iran, Tehran, Iran; 2 Department of Pathobiology, School of Public Health, Tehran University of Medical Sciences, Tehran, Iran; 3 Microbiology Research Center (MRC), Pasteur Institute of Iran, Tehran, Iran; University of Bari: Universita degli Studi di Bari Aldo Moro, ITALY

## Abstract

**Background:**

*Enterobacter* is a major nosocomial genus of *Enterobacteriaceae* responsible for a variety of nosocomial infections, particularly in prolonged hospitalized patients in the intensive care units. Since current antibiotics have failed treating colistin- and carbapenem-resistant *Enterobacteriaceae*, efforts are underway to find suitable alternative strategies. Therefore, this study conducted a reverse vaccinology (RV) to identify novel and putative immunogenic targets using core proteome of 20 different sequence types (STs) of clinical *Enterobacter* spp. Moreover, we introduced a structural-based approach for exploration of potential vaccine candidates against the *Enterobacteriaceae* family using their conserved domain analysis.

**Results:**

A number of 2616 core coding sequences (CDSs) were retrieved from 20 clinical strains of *Enterobacter* spp. with a similarity of ≥ 50%. Nine proteins with a score of ≥ 20 considered as the shortlisted proteins based on the quartile scoring method, including three TonB-dependent receptors, WP_008500981.1, WP_058690971.1 and WP_058679571.1; one YjbH domain-containing protein, WP_110108068.1; three flagellar proteins, WP_088207510.1, WP_033145204.1 and WP_058679632.1; one spore-coat U domain-containing protein, WP_039266612.1; and one DD-metalloendopeptidase family protein, WP_025912449.1. In this study, proteins WP_058690971.1 and WP_110108068.1 were detected as the top candidates with regard to immune stimulation and interactions with TLRs. However, their efficacy is remaining to be evaluated experimentally.

**Conclusions:**

Our investigation introduced common ferrichrome porins with high sequence similarity as potential vaccine candidates against the *Enterobacteriaceae* family. These proteins belong to the iron acquisition system and possess all criteria of suitable vaccine targets. Therefore, they need to be specifically paid attention for vaccine development against clinically important members of *Enterobacteriaceae* family.

## Introduction

*Enterobacter* is a genus of common Gram-negative, facultatively anaerobic, rod-shaped, non-spore-forming, and motile bacteria belonging to the *Enterobacteriaceae* family. There are 22 highly similar species within this genus, comprising *Enterobacter cloacae* complex (ECC) [1, 2]. Some ECC strains originate from soil and water, and some are natural commensals of the animal and human gut. However, they are capable of causing a variety of infections such as respiratory tract infections, urinary tract infections (UTIs), soft-tissue infections, septicemia, and meningitis with severe medical sequela [3]. Among members of this complex, *E*. *cloacae*, *E*. *hormaechei*, *E*. *kobei*, and *E*. *roggenkampii* are frequently isolated from clinical specimens. *E*. *hormaechei* and *E*. *kobei* account for more than 70% of community-acquired infections. *E*. *cloacae* is responsible for 10% of post-surgical peritonitis and four to five percent of nosocomial sepsis, pneumonia cases, and UTIs [4]. These species have wide geographical distribution all over the world, frequently reported from Australia, the United States, Germany, and China [5].

The treatment of ECC infections is problematic due to the increasing resistance to various antimicrobial agents [6]. Recent studies have shown that *Enterobacter* spp. are often the second or the third most common nosocomial *Enterobacteriaceae* harboring carbapenemase enzymes [7]. Therefore, there is an urgent need to eliminate such highly resistant bacteria via new options other than antibiotics. With this regard, various effective strategies can be introduced. Among them, vaccination is promising as it represents microorganism-specific prevention, confining the spread of infection and reducing clinical manifestations, drug side effects, patient hospitalization as well as average medical expenses [8, 9]. However, despite the highly frequent and extended antimicrobial resistance of ECC, no effective vaccine has been developed so far.

The ability of conventional methods to discover immunological compounds of microorganisms is limited [10]. The application of whole genomic data along with computational bioinformatics enables us to explore proteins for vaccine design. Reverse vaccinology (RV) is a desirable method because it computationally identifies vaccine candidates, the majority of which cannot be detected through wet-lab experiments [11]. A few studies have been conducted on *in silico* identification of novel putative vaccine candidates against ECC up to now. In 2020, three outer-membrane porin proteins, including LpfC, OmpA, and FimD as well as an arginine transporter were introduced using a subtractive study of *E*. *cloacae* reference proteome [12]. More recently, Alshammari *et al*., used bioinformatics to design a multi-chimeric vaccine against *E*. *xiangfangensis* using the ferrichrome porin (FhuA) and peptidoglycan-associated lipoprotein (Pal) [13]. Also, Phosphoporin E (PhoE) and a putative outer-membrane porin protein presented appropriate characteristics for vaccine design [14].

In the present study, we aimed to introduce novel putative immunogenic candidates against different sequence types (STs) of clinical ECC strains using their core proteome. In addition, we investigated consensus protein structures with promising immunogenicity against other clinically important *Enterobacteriaceae*.

## Materials and methods

### Initial protein screening

#### Selection of *Enterobacter* spp. Strains

The dataset included almost all *E*. cloacae complex causing human clinical infections isolated from various clinical samples (not animal/environmental sources), different geographical regions and different periods of time (from 2008 to 2020). Please see S1 Table. We picked up 20 *Enterobacter* spp. belonging to seven species including *E*. *cloacae*, *E*. *hormaechei*, *E*. *kobei*, *E*. *roggenkampii*, *E*. *cancerogenus*, *E*. *asburiae*, and *E*. *bugandensis*, considering all the above criteria for our analysis. The complete genomes of selected ECC strains were retrieved from the NCBI database (https://www.ncbi.nlm.nih.gov/). The genetic characteristics and clinical information of strains have been provided in S1 Table.

#### Genetic and phylogenetic comparison of ECC strains

PubMLST (https://pubmlst.org/) was used to determine strains’ STs based on the allele numbers of six housekeeping genes (*rpoB*, *fusA*, *gyrB*, *leuS*, *pyrG*, and *rplB*). PubMLST is a collection of integrated population sequence data, containing the provenance, phylogenetic and phenotypic information for over 100 different microbial species and genera [15]. To compare the strains on the whole genomic level, multiple circular alignment was performed using the BLAST Ring Image Generator (BRIG) software version 0.95. BRIG is a free application that can visualize similarities and differences of genomes and compare their genetic features [16]. In this study, *E*. *cloacae* subsp. *cloacae* ATCC 13047 (accession number: NC_014121.1) was used as the reference strain. To elucidate their phylogenetic distances, a Neighbor Joining (NJ) dendrogram based on the core genome multi-locus sequence typing (cg-MLST) was performed using cano-wgMLST_BacCompare (http://baccompare.imst.nsysu.edu.tw). This server enables users to determine the evolutionary relationship of bacteria using a whole genome (wg-) MLST and canonical MLST [17]. The number, distribution, and functional classification of pan, core, accessory and unique coding sequences (CDSs) of 20 ECC strains were achieved by the Bacterial Pan Genome Analysis (BPGA) tool [18]. Finally, the core proteome was obtained using BPGA with an identity cut-off value of ≥ 50%.

#### Prediction of subcellular localization of proteins

All proteins were uploaded to PSORTb (www.psort.org/psortb/) as a database to predict the subcellular localization of proteins [19]. Only cell wall, extracellular, secreted, and surface-exposed proteins were selected. The topology of the proteins was predicted using the HMMTOP (http://www.enzim.hu/hmmtop/) database which is an automatic server for predicting transmembrane helices and the topology of proteins [20].

#### Antigenicity and allergenicity determination of proteins

The antigenicity of the proteins was predicted with the VaxiJen online tool (http://www.ddg-pharmfac.net/vaxijen/VaxiJen/VaxiJen.html) using a cut-off value of ≥ 0.5. VaxiJen is a server for alignment-independent prediction of protective antigens [21]. Subsequently, the allergenicity of the antigenic proteins was determined using the AlgPred 2.0 tool (https://webs.iiitd.edu.in/raghava/algpred2/batch.html) with a cut-off value of ≥ 0.5. This web tool has been developed for prediction of amino acid residues with allergenic characteristics in a protein sequence [22].

#### Sequence similarity of putative immunogenic targets with the human proteome

PSI-BLAST (https://blast.ncbi.nlm.nih.gov) with the defined threshold (coverage ≥ 30% and identity ≥ 25%) was used to assess the homology of selected proteins versus human proteome (*Humo sapiens*, taxid: 9606) [23, 24]. PSI-BLAST provides a BLASTp search with a custom, position-specific, scoring matrix which can help to find distant evolutionary relationships [25].

### Comparative analysis

#### Characterization of physiochemical properties, functional class and adhesin probability

The molecular weight and other physiochemical properties of the selected proteins such as theoretical pI, half-life, instability and hydropathy indexes were estimated using the ProtParam tool (https://web.expasy.org/protparam/). This web tool computes various physical and chemical parameters for a particular protein [26]. The adhesion probability and functional class of the proteins were determined using the Vaxign (http://www.violinet.org/vaxign2) and VICMpred (http://www.imtech.res.in/raghava/vicmpred/) databases, respectively. Vaxign predicts adhesion probability of proteins using an optimized SPAAN. It has been approved that the SPAAN prediction has a sensitivity of 89% and specificity of 100% [27]. VICMpred provides a SVM-based method to stimulate functional classification of bacterial proteins and classifies them into virulence factors, information molecules, cellular processes and metabolic molecules [28].

#### Determination of linear B-cell epitopes and human MHC II binding sites

The BepiPred v2.0 tool (http://www.cbs.dtu.dk/services/BepiPred/) was used to identify linear B-cell epitopes of all previously selected proteins with a threshold of ≥ 0.61. This tool uses a random forest algorithm trained on epitopes of antibody-antigen protein structures [29]. For each protein, the B-cell epitopes’ ratio was calculated (the proportion of the number of amino acids in B-cell epitopes to the total number of amino acids of each protein). Next, TepiTool (http://tools.iedb.org/tepitool/) from the Immune Epitope Database (IEDB) resource, was used to predict human MHC II binding sites (T-cell epitopes) with a cut-off value of the top 5% of peptides. The ratio of MHC II binding sites was calculated for each protein (the proportion of binding sites to the total amino acids of each protein). This database uses experimental data on antibody and T-cell epitopes studied in humans, non-human primates, and other animal species in relation to infectious diseases, autoimmunity, allergies, and transplantation [30].

#### Prediction of conformational B-cell epitopes

In this section, the tertiary structures of the putative immunogenic candidates were first predicted using the Robetta web tool (https://robetta.bakerlab) [31]. The quality of each tertiary structure prediction was checked using QMEAN (https://swissmodel.expasy.org/qmean/) and the ProSA-web server (https://prosa.services.came.sbg.ac.at/prosa.php). The QMEAN server provides the quality estimation of protein structure models by ranking potentially unreliable regions within them [32]. ProSA-web server is useful for the recognition of errors in three-dimensional (3D) structures of proteins [33].

Next, the conformational B-cell epitopes of selected proteins were identified using the IEDB analysis resource (http://tools.iedb.org/ellipro/) with a threshold value of ≥ 0.8 [34]. The tertiary structures of proteins were visualized using PyMOL version 2.3.4 (Schrödinger, LLC.). PyMOL is a user-sponsored molecular visualization system on an open-source foundation.

### Shortlist selection based on the quartile scoring method

The quartile method is based on the evaluation of eight properties including adhesion probability, antigenicity index, hydropathy index, instability index, functional class (virulence, cellular process, information and storage, and metabolic molecule), B-cell epitopes ratio, T-cell epitopes ratio, and the number of conformational B-cell epitopes. The sum of all scores for each protein was considered in the final score. Proteins with a score of ≥ 20 were considered putative immunogenic proteins and underwent further analysis. The quartile method measures the dispersion of values by dividing their distribution into lower, median, and upper quartiles to form four intervals. The quartile method provides us a rational target selection by comparing proteins based on several unweighted criteria at the same time [35].

### Immune simulation and molecular docking

The immune-reactivity of the shortlisted proteins were simulated using C-ImmSim (https://kraken.iac.rm.cnr.it/C-IMMSIM/index.php). C-ImmSim utilizes a position-specific scoring matrices (PSSM) originated from machine learning techniques to simulate immunological reactions [36]. C-ImmSim also predicts the anatomical regions where crucial events of immunity occur [37]. The number of injections was considered 1 time without LPS for each immunogenic candidate. The protein-protein rigid docking was also performed for the prediction of their binding affinity to surface-exposed human TLR-1, 2, and 4 using pyDockWEB (https://life.bsc.es/pid/pydockweb/default/index). This is a web-server for the prediction of protein-protein rigid interactions [38].

### Conserved domains analysis

The conserved domain database, CDD (https://www.ncbi.nlm.nih.gov/Structure/cdd/cdd.shtml), was used for prediction of taxonomic relationships of proteins and their functions based on their conserved domains [39]. In addition, the best putative candidates were investigated for their similar proteins in other genera of the *Enterobacteriaceae* family using this web tool.

## Results

### Comparative genomic analysis of ECC strains and their phylogenetic relationship

**Fig 1:**
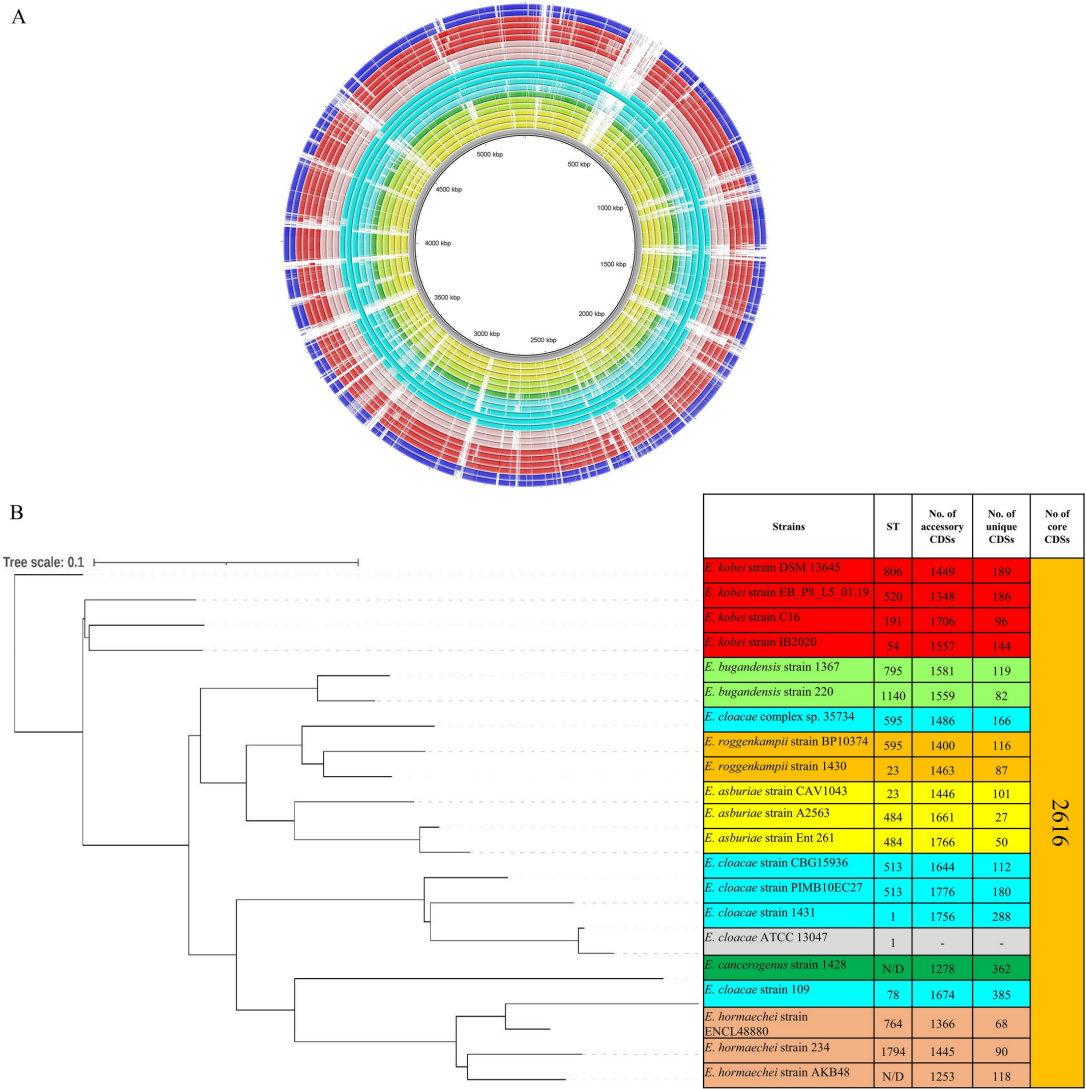
A. The whole genomic multiple sequence alignment of 20 different clinical *Enterobacter* spp. isolates. The results showed their high similarity. B. The phylogenetic dendrogram based on cg-MLST using 1680 genes with ≥ 95% sequence similarity. The results indicated a close ancestral relationship between different members of *Enterobacter* spp. A number of 2616 core coding sequences were retrieved from their genome with ≥ 50% similarity.

The multiple sequence alignment of selected clinical *Enterobacter* spp. showed high similarity of strains at the whole genomic level. See Fig 1A. The strains belonged to different STs including 513, 484, 595, 795, 764, 54, 520, 78, 806, 1, 23 191, 1140 and 1794. See Fig 1B. The phylogenetic dendrogram based on 1680 genes with ≥ 95% sequence similarity indicated a close ancestral relationship between different members of ECC. Strains belonging to each single *Enterobacter* spp. were located close in consensus nodes except for *E*. *cloacae* complex sp. 35734.

During core proteome analysis, a number of 2616 core CDSs were found among 20 clinical strains of *Enterobacter* spp. with ≥ 50% sequence similarity. The lowest and highest number of accessory CDSs were for *E*. *hormaechei* strain AKB48 (1253) and *E*. *cloacae* strain PIMB10EC27 (1776), respectively. In addition, *E*. *asburiae* strain A2563 (27 CDSs) and *E*. *cancerogenus* strain 1428 (362 CDSs) had the minimum and maximum numbers of unique CDSs, respectively. See Fig 1B.

**Fig 2:**
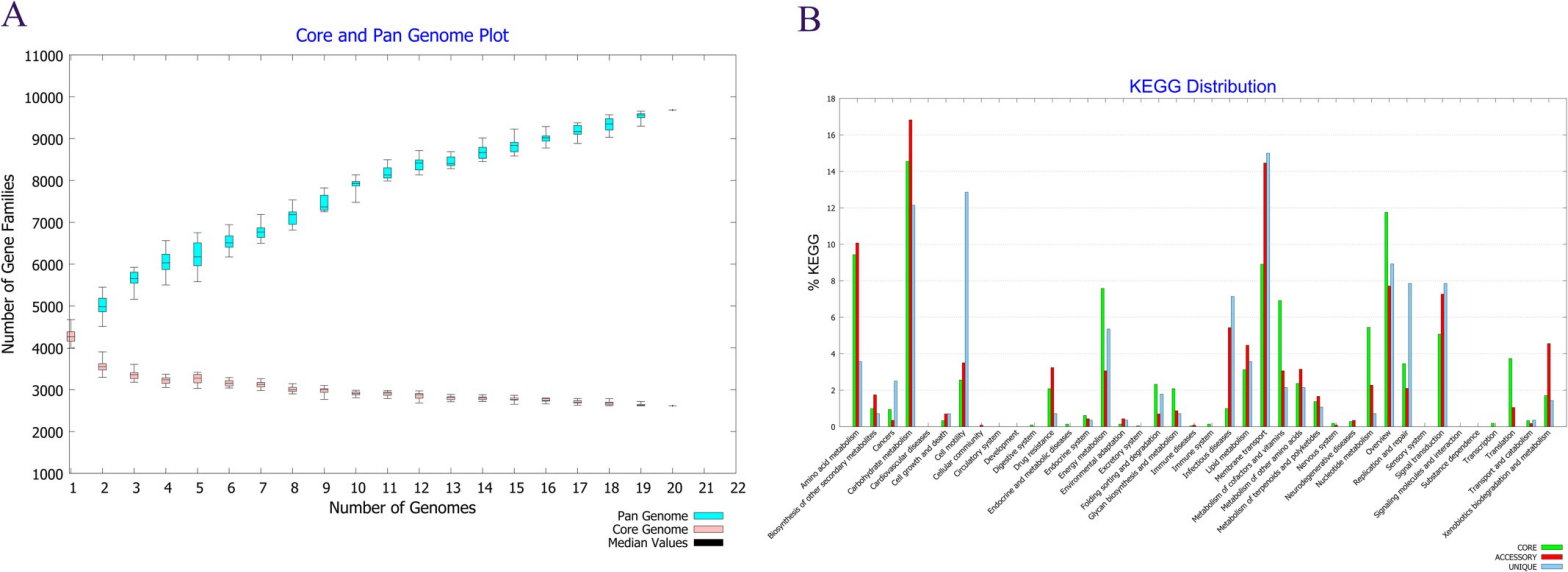
A. The distribution of pan/core gene families of 20 clinical *Enterobacter* species. The pan/core gene profiling of ECC strains indicated that the number of pan gene families differs meaningfully among the strains. While, the distribution of core gene families was almost close. B. The functional classification of core, accessory and unique genes among 20 clinical *Enterobacter* species. The orthologous core, accessory and unique genes were clustered in 19 known functional classes and one unknown class.

The pan/core gene profiling of ECC strains indicated that the number of pan gene families differs meaningfully among the strains. While, the distribution of core gene families was almost close. See Fig 2A. The core, accessory and unique orthologous genes were clustered in 19 known functional classes and one unknown class. See Fig 2B. The majority of core CDSs were involved in general functions as well as amino acid metabolism/transportation while of them very few were associated with intercellular trafficking and defense mechanisms. The majority of accessory genes were related to general functions and transcription. The majority of unique CDSs were involved in general functions, replication/repair as well as transcription. The fewest percentage of accessory and unique genes were involved in nucleotide transport/metabolism, translation and ribosomal biogenesis.

### Subtracted proteins

**Fig 3:**
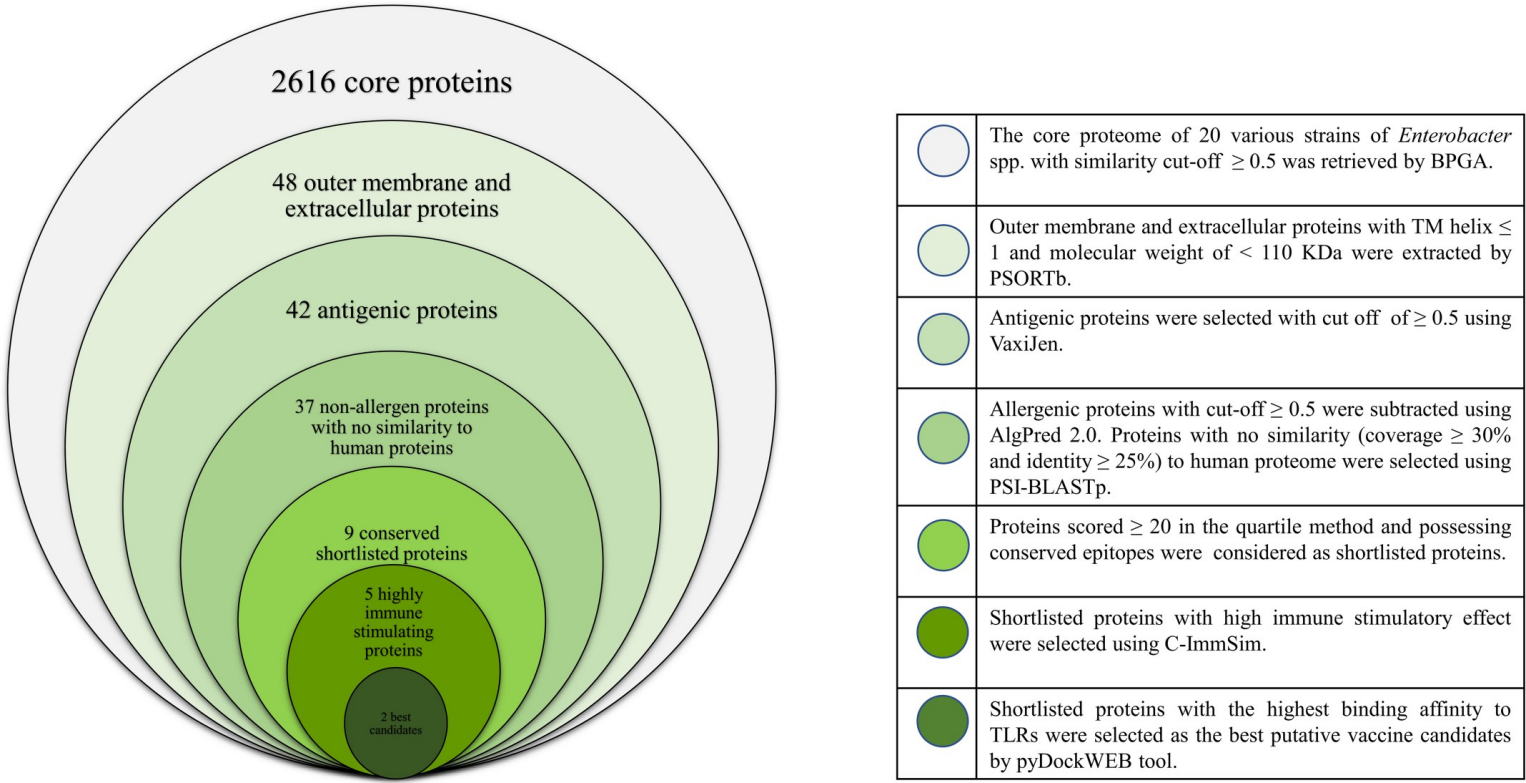
The workflow used for the identification of putative immunogenic candidates against *E*. *cloacae* complex.

Among 2616 core proteins, only 48 proteins were outer membrane or extracellular. The number of the transmembrane helices in all proteins were ≤ 1 and no proteins weighed >110 kDa. Among them, 42 proteins were antigenic and five were allergenic. Overall, 37 antigenic, non-allergen proteins with no similarity to human proteins were subtracted for comparative analysis. The flowchart summarizing step by step subtraction of vaccine candidates has been shown in Fig 3.

### Shortlisted proteins based on the quartile scoring method

**Fig 4:**
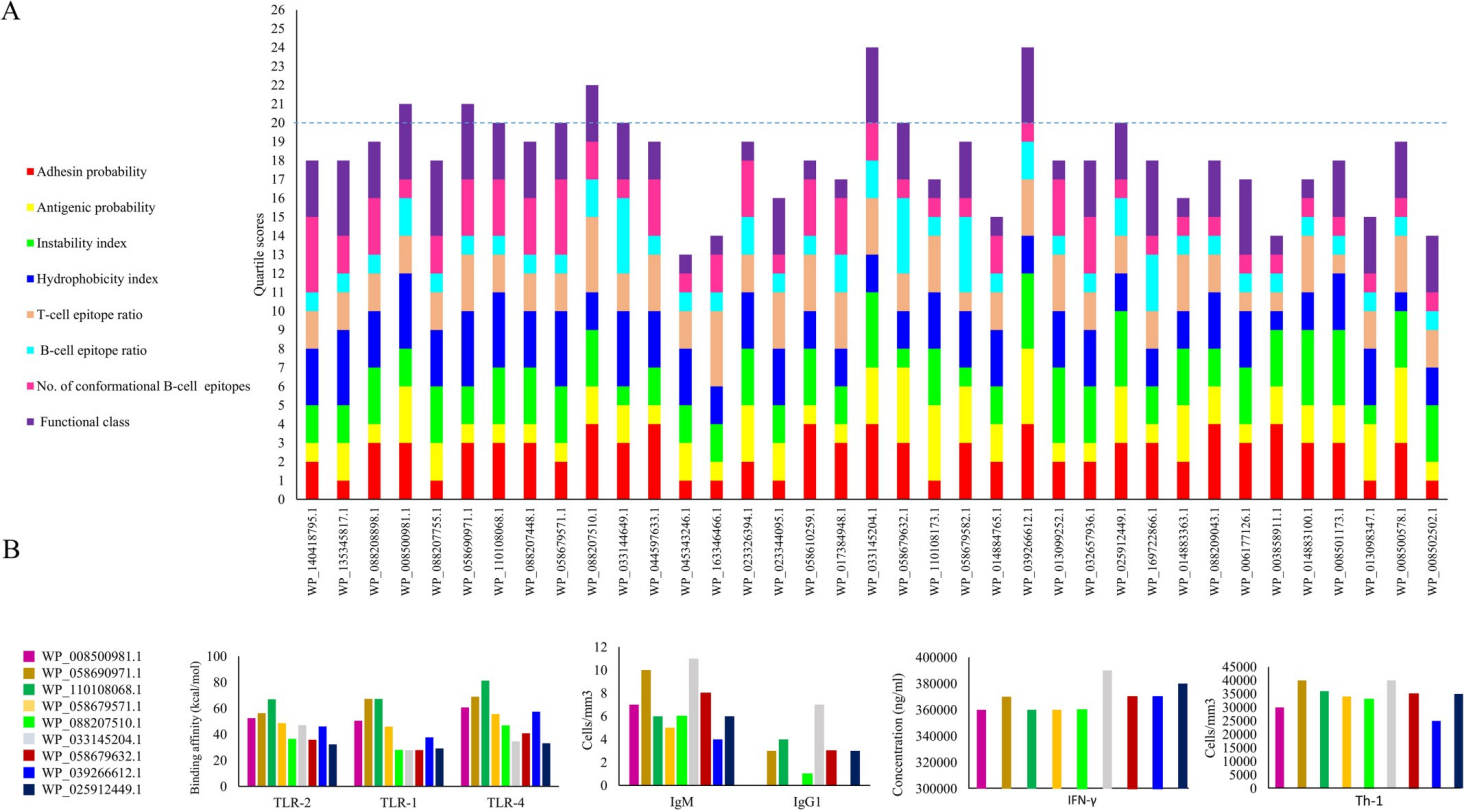
A. The comparison of 37 proteins of *E*. *cloacae* complex based on the quartile method. Nine proteins with a score of ≥ 20 were considered as the shortlisted proteins based on the following eight properties: adhesion probability, antigenicity index, hydropathy index, instability index, functional class (virulence, cellular process, information and storage, and metabolic molecule), B-cell epitopes ratio, T-cell epitopes ratio, and the number of conformational B-cell epitopes. B. The comparison of immunological responses induced by nine shortlisted vaccine candidates against *E*. *cloacae* complex as well as their binding affinity to TLRs. The results showed that five proteins including WP_058690971.1, WP_110108068.1, WP_033145204.1, WP_058679632.1 and WP_025912449.1 could stimulate the production of IFN-γ, IgM, IgG1. Among these, WP_058690971.1 and WP_110108068.1 had the highest binding affinity to TLR-1, 2 and 4.

Nine proteins with a score of ≥ 20 were considered as the shortlisted proteins based on eight properties, including adhesion probability, antigenicity index, hydropathy index, instability index, functional class (virulence, cellular process, information and storage, and metabolic molecule), B-cell epitopes ratio, T-cell epitopes ratio, and the number of conformational B-cell epitopes. See Fig 4A. The shortlisted putative candidates were as follows: five outer membrane proteins including WP_008500981.1 (TonB-dependent siderophore receptor), WP_058690971.1 (TonB-dependent siderophore receptor), WP_058679571.1 (TonB-dependent vitamin B12 receptor BtuB) and WP_110108068.1 (YjbH domain-containing protein); WP_025912449.1 (peptidoglycan DD-metalloendopeptidase family protein); and four extracellular proteins including WP_088207510.1 (flagellar hook-associated protein FlgK), WP_033145204.1 (flagellar hook protein FlgE), WP_058679632.1 (flagellar hook length control protein FliK), and WP_039266612.1 (spore-coat U domain-containing protein). See Fig 4A.

### Comparison of the shortlisted proteins

**Fig 5:**
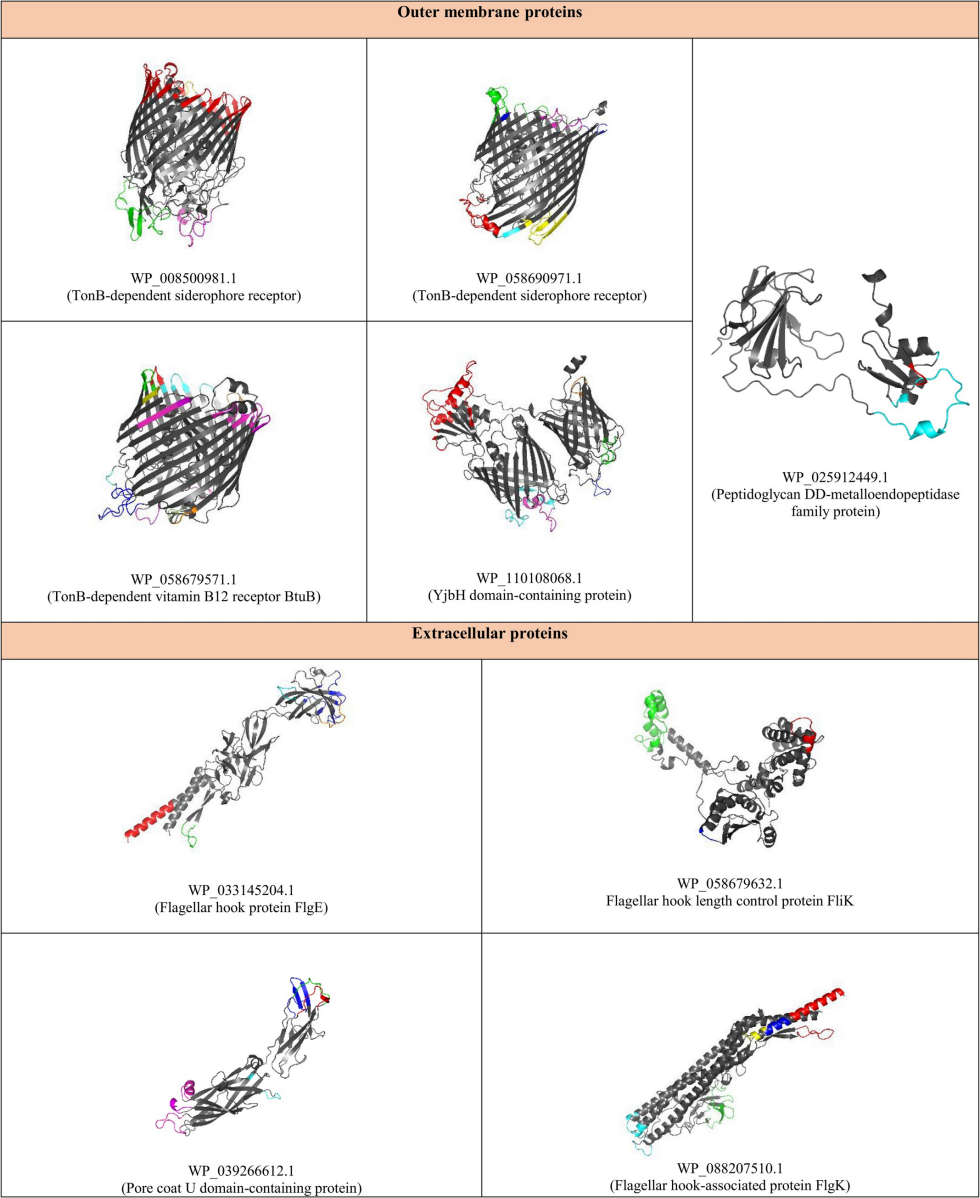
The tertiary structure and conformational B-cell epitopes of nine shortlisted proteins against *E*. *cloacae* complex. Nine proteins with a score of ≥ 20 were subtracted. The shortlisted putative candidates were as follows: five outer membrane proteins including WP_008500981.1 (TonB-dependent siderophore receptor), WP_058690971.1 (TonB-dependent siderophore receptor), WP_058679571.1 (TonB-dependent vitamin B12 receptor BtuB) and WP_110108068.1 (YjbH domain-containing protein); WP_025912449.1 (peptidoglycan DD-metalloendopeptidase family protein); and four extracellular proteins including WP_088207510.1 (flagellar hook-associated protein FlgK), WP_033145204.1 (flagellar hook protein FlgE), WP_058679632.1 (flagellar hook length control protein FliK), WP_039266612.1 (spore-coat U domain-containing protein).

Molecular weights of proteins ranged from 26.13 to 82.87 kDa. All of them were predicted to be stable. WP_033145204.1 has the lowest instability index (14.73). TonB-dependent siderophore receptors, FlgE and FliK were virulence factors while other remaining proteins were involved in cellular processes. WP_008500981.1 and WP_025912449.1 had the maximum and minimum number of linear B-cell epitopes, respectively. The B-cell epitope ratio ranged from 0.10 (for WP_058690971.1) to 0.51 (for WP_058679632.1). WP_058690971.1 and WP_025912449.1 had the highest and lowest number of T-cell epitopes, respectively. WP_088207510.1 had the maximum T-cell epitope ratio (0.15). The number of conformational B-cell epitopes ranged from two (for WP_025912449.1) to eight (for WP_058679571.1). Physicochemical properties, functional class, number of linear and conformational B-cell epitopes, MHC II binding sites of shortlisted proteins have been presented in S2 Table. The tertiary structure of shortlisted proteins as well as their conformational B-cell epitopes have been depicted in Fig 5.

### Immune simulation and molecular docking

**Fig 6:**
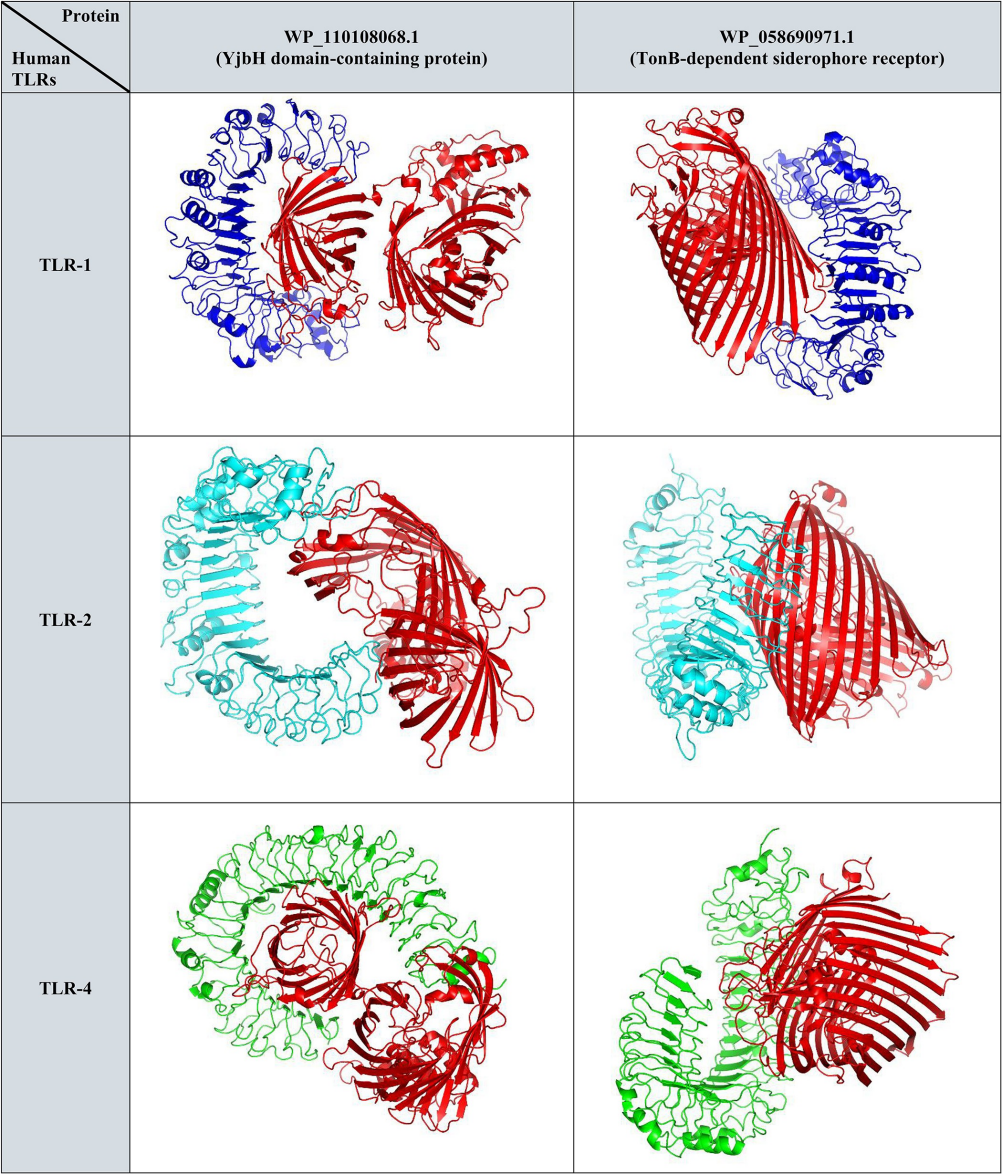
The interaction of WP_058690971.1 (TonB-dependent siderophore receptor) and WP_110108068.1 (YjbH domain-containing protein) with TLR-1, 2 and 4.

The results of the immune simulation showed that five proteins including WP_058690971.1, WP_110108068.1, WP_033145204.1, WP_058679632.1 and WP_025912449.1 could stimulate the production of IFN-γ, IgM and IgG1. See Fig 4B and Table 1. Among them, WP_058690971.1 and WP_110108068.1 had the highest binding affinity to TLR-1, 2 and 4. The interaction of these two proteins with TLR-1, 2 and 4 has been presented in Fig 6. Considering both criteria, WP_058690971.1 and WP_110108068 were selected as the best vaccine candidates against the clinical *Enterobacter* spp. The results of immune simulation and molecular docking have been summarized in Table 1.

**Table 1:** Results of immune simulations and molecular dockings of nine putative vaccine candidates against clinical *Enterobacter* spp.

Immune response prediction Vaccine candidates	Immune simulations				Binding affinities		
	IFN-γ	IgM	IgG1	Th1	TLR-2	TLR-1	TLR-4
	(ng/ml)	(cells/mm^3^)	(cells/mm^3^)	(cells/mm^3^)	(kcal/mol)	(kcal/mol)	(kcal/mol)
**WP_008500981.1**	360000	7	0	30000	-52.427	-50.506	-60.703
**(TonB-dependent siderophore receptor)**							
**WP_058690971.1**	**370000**	**10**	**3**	**40000**	**-56.349**	**-67.264**	**-68.984**
**(TonB-dependent siderophore receptor)**							
**WP_110108068.1**	**360000**	**6**	**4**	**36000**	**-66.994**	**-67.264**	**-81.345**
**(YjbH domain-containing protein)**							
**WP_058679571.1**	360000	5	0	34000	-48.639	-45.921	-55.599
**(TonB-dependent vitamin B12 receptor BtuB)**							
**WP_088207510.1**	360000	6	1	33000	-36.503	-28.029	-46.974
**(Flagellar hook-associated protein FlgK)**							
**WP_033145204.1**	**390000**	**11**	**7**	**40000**	-47.016	-27.741	-34.604
**(Flagellar hook protein FlgE)**							
**WP_058679632.1**	**370000**	**8**	**3**	**35000**	-35.844	-27.842	-40.801
**(Flagellar hook length control protein FliK)**							
**WP_039266612.1**	370000	4	0	25000	-46.123	-37.685	-57.411
**(Pore coat U domain-containing protein)**							
**WP_025912449.1**	**380000**	**6**	**3**	**35000**	-32.314	-29.089	-33.082
**(Peptidoglycan DD-metalloendopeptidase family protein)**							

*Bold parameters indicate values above the mean.

### Conserved domains

The results obtained from CDD demonstrated that nine shortlisted proteins have superfamilies with three main functions: iron uptake (WP_008500981.1, WP_058690971.1, and WP_058679571.1), exopolysaccharide production (WP_110108068.1) and flagellar assembly (WP_088207510.1, WP_033145204.1, and WP_058679632.1). WP_039266612.1 had a spore-coat U domain involved in spore coating. WP_025912449.1 had domains responsible for peptidoglycan hydrolase (NlpD, M23 peptidase and LysM). The full results of the CDD search have been summarized in Table 2.

**Table 2:** Subcellular localization, conserved domains, related taxonomy and functions of nine putative vaccine candidates against clinical *Enterobacter* spp.

Protein Accession Number	Subcellular Localization	Conserved Domain	Domain taxonomy	Function
**WP_008500981.1**	Outer Membrane	FepA family TonB-dependent siderophore receptor	*Enterobacteriaceae*	TonB-dependent siderophore receptor acts as a channel to allow import of iron-siderophore complexes, such as *Escherichia coli* ferrienterobactin receptor, which is involved in the initial step of iron uptake by binding ferrienterobactin.
**(TonB-dependent siderophore receptor)**				
		FepA	Proteobacteria	FepA is the outer membrane receptor for ferrienterochelin and colicins [Inorganic ion transport and metabolism].
		Ligand_gated_channel	Bacteria	TonB dependent/Ligand-Gated channels are created by a monomeric 22 strand (22, 24) anti-parallel beta-barrel. Ligands apparently bind to the large extracellular loops. The N-terminal 150–200 residues form a plug from the periplasmic end of barrel. Energy (proton-motive force) and TonB-dependent conformational alteration of channel (parts of plug, and loops 7 and 8) allow passage of ligand. FepA residues 12–18 form the TonB box, which mediates the interaction with the TonB-containing inner membrane complex. TonB preferentially interacts with ligand-bound receptors. Transport thru the channel may resemble passage thru an air lock. In this model, ligand binding leads to closure of the extracellular end of pore, then a TonB-mediated signal facilitates opening of the interior side of pore, deforming the N-terminal plug and allowing passage of the ligand to the periplasm. Such a mechanism would prevent the free diffusion of small molecules.
		TonB_dep_Rec	Bacteria	TonB-dependent siderophore receptor; This subfamily model encompasses a wide variety of TonB-dependent outer membrane siderophore receptors. It has no overlap with TonB receptors known to transport other substances, but is likely incomplete due to lack of characterizations. It is likely that genuine siderophore receptors will be identified which score below the noise cutoff to this model at which point the model should be updated. [Transport and binding proteins, Cations and iron carrying compounds, Transport and binding proteins, Porins].
**WP_058690971.1**	Outer Membrane	Ferrichrome outer membrane transporter	*Enterobacteriaceae*	-
**(TonB-dependent siderophore receptor)**				
		Ligand_gated_channel	Bacteria	-
		TonB-siderophor	Bacteria	TonB-dependent siderophore receptor; This subfamily model encompasses a wide variety of TonB-dependent outer membrane siderophore receptors. It has no overlap with TonB receptors known to transport other substances, but is likely incomplete due to lack of characterizations. It is likely that genuine siderophore receptors will be identified which score below the noise cutoff to this model at which point the model should be updated. [Transport and binding proteins, Cations and iron carrying compounds, Transport and binding proteins, Porins].
		CirA	Bacteria	Outer membrane receptor proteins, mostly Fe transport [Inorganic ion transport and metabolism].
		TonB_dep_Rec	Bacteria	-
**WP_110108068.1**	Outer Membrane	YjbH	Bacteria	YjbH domain-containing protein, similar to *Escherichia coli* K-12 YjbH which is a putative lipoprotein and/or porin involved in exopolysaccharide production. It is an exopolysaccharide biosynthesis protein. YjbH is a family of Gram-negative beta-barrel.
**(YjbH domain-containing protein)**				
**WP_058679571.1**	Outer Membrane	BtuB	*Enterobacteriaceae*	TonB-dependent vitamin B12 receptor BtuB is involved in the active translocation of vitamin B12 (cyanocobalamin) across the outer membrane to the periplasmic space.
**(TonB-dependent vitamin B12 receptor BtuB)**				
		TonB-B12	Gammaproteobacteria	TonB-dependent vitamin B12 receptor; This model represents the TonB-dependent outer membrane receptor found in Gammaproteobacteria responsible for translocating the cobalt-containing vitamin B12 (cobalamin). [Transport and binding proteins, Other, Transport and binding proteins, Porin].
		TonB_sider_MxcH	Bacteria	TonB-dependent siderophore myxochelin receptor MxcH.
		Ligand_gated_channel	Bacteria	-
		TonB_dep_Rec	Bacteria	-
**WP_088207510.1**	Extracellular	FlgK	Proteobacteria	Flagellar hook-associated protein FlgK forms the junction between the hook and the filament in the flagellum together with FlgL and provides a structural base where flagellin, a filament-forming protein, is inserted for the initiation of filament elongation.
**(Flagellar hook-associated protein FlgK)**				
		FlgK_ends	Bacteria	Flagellar hook-associated protein FlgK; The flagellar hook-associated protein FlgK of bacterial flagella has conserved N- and C-terminal domains. The central region is highly variable in length and sequence, and often contains substantial runs of low-complexity sequence. This model is built from an alignment of FlgK sequences with the central region excised.
		Flagellar basal body rod FlgEFG protein C-terminal	Bacteria	This family consists of a number of C-terminal domains of unknown function. This domain seems to be specific to flagellar basal-body rod and flagellar hook proteins in which pfam00460 is often present at the extreme N-terminus.
**WP_033145204.1**	Extracellular	FlgE	Bacteria	Flagellar hook protein FlgE functions as a nano-sized universal joint, which is essential for dynamic and efficient bacterial motility and taxis.
**(Flagellar hook protein FlgE)**				
		Flagellar basal body rod FlgEFG protein C-terminal	Bacteria	This family consists of a number of C-terminal domains of unknown function. This domain seems to be specific to Flagellar basal-body rod and flagellar hook proteins in which pfam00460 is often present at the extreme N-terminus.
**WP_058679632.1**	Extracellular	PRK10118	*Enterobacteriaceae*	Flagellar hook-length control protein FliK controls elongation by determining hook length and by stopping the supply of hook protein to the filament protein.
**(Flagellar hook length control protein FliK)**				
		C-terminal domain of flagellar hook-length control protein FliK and similar domains;	Cellular organisms	The flagellar hook-length control protein FliK is a soluble cytoplasmic protein that is secreted during Flagellar formation. It controls hook elongation by two successive events: by determining hook length and by stopping the supply of hook protein. It contains an N-terminal domain that determines hook length and a C-terminal domain that is responsible for switching secretion from the hook protein to that of the filament protein, by interacting with FlhB, the switchable secretion gate.
		Flg_hook	Cellular organisms	It controls the length of the Flagellar hook by directly measuring the hook length as a molecular ruler. This family also includes YscP of the *Yersinia* type III secretion system, and equivalent proteins in other pathogenic bacterial type III secretion systems.
**WP_039266612.1**	Extracellular	SCPU	Proteobacteria	This domain is found in a bacterial family of spore coat proteins. Spore coat U (SCPU) domain-containing protein may act as a bacterial spore coat protein or as a secreted pili protein subunit involved in motility and biofilm formation; the family is distantly related to fimbrial proteins
**(Spore coat U domain-containing protein)**				
**WP_025912449.1**	Outer Membrane	NlpD	Enterobacteriaceae	Murein hydrolase activator NlpD is the activator of the cell wall hydrolase AmiC. It is Required for septal murein cleavage and daughter cell separation during cell division.
**(Peptidoglycan DD-metalloendopeptidase family protein)**				
		Peptidase_M23	Bacteria	Members of this family are zinc metallopeptidases with a range of specificities. The peptidase family M23 is included in this family, these are Gly-Gly endopeptidases. Peptidase family M23 are also endopeptidases. This family also includes some bacterial lipoproteins for which no proteolytic activity has been demonstrated. This family also includes leukocyte cell-derived chemotaxin 2 (LECT2) proteins. LECT2 is a liver-specific protein which is thought to be linked to hepatocyte growth although the exact function of this protein is unknown.
		LysM	Bacteria	-

### Evaluation of TonB-dependent siderophore receptors in other members of *Enterobacteriaceae* as putative vaccine candidates

**Fig 7:**
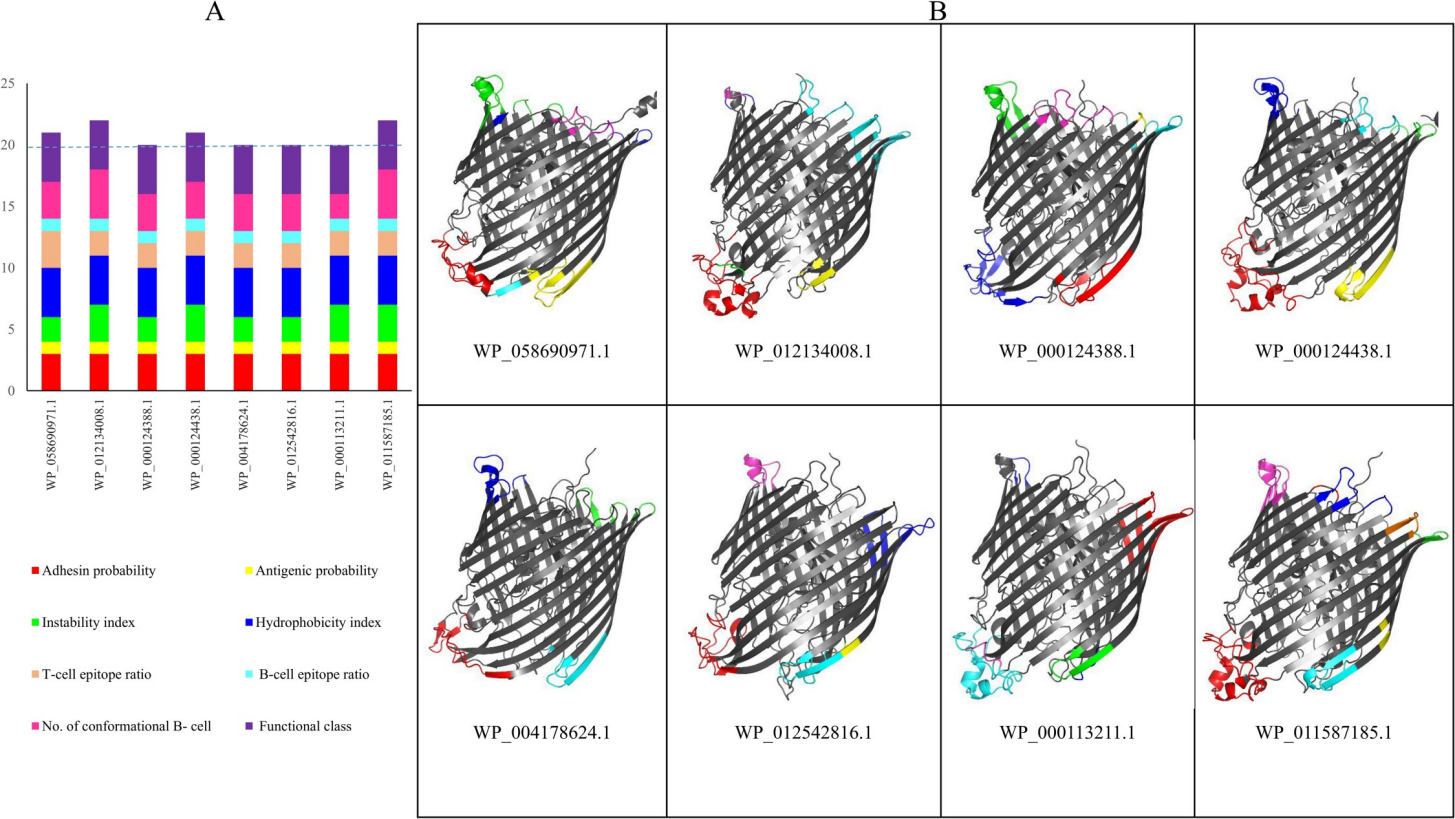
A. The comparison of eight proteins belonging to ferrichrome outer membrane transporter conserved superfamily in eight clinically important genera of *Enterobacteriaceae*. All selected proteins scored ≥ 20 based on the quartile method with physicochemical properties, functional class, number of linear and conformational B/T-cell epitopes similar to our candidate, TonB-dependent siderophore receptor (WP_058690971.1). B. The tertiary structure and conformational B-cell epitopes of ferrichrome porins in major clinically important genera of *Enterobacteriaceae* family.

We identified the conserved domain of the ferrichrome outer membrane transporter superfamily in WP_058690971.1 (TonB-dependent siderophore receptor). This superfamily exists in ferrichrome porin FhuA of all members of *Enterobacteriaceae*. We detected this ferrichrome porin in major clinically important *Enterobacteriaceae* including *Escherichia coli* (WP_000124438.1 and WP_000124388.1) *Klebsiella pneumoniae* (WP_004178624.1 and WP_012542816.1), *Salmonella enterica* (WP_000113211.1), *Shigella flexneri* (WP_011587185.1) and *Citrobacter koseri* (WP_012134008.1). All selected proteins scored ≥ 20 based on the quartile method. See Fig 7A. The results of immune simulation and molecular docking for all seven proteins were similar to that of our candidate. See S3 Table. The tertiary structures of *Enterobacteriaceae* ferrichrome porins have been presented in Fig 7B. In addition, all information regarding these proteins have been shown in S3 Table.

## Discussion

This study utilized the core proteome of various *Enterobacter* spp. to screen several immunogenic candidates in the first place. The results of cg-MLST indicated a close phylogenetic relationship among selected strains on the core genomic level and the number of their core CDSs were also in the same range. Therefore, by excluding the accessory genetic content; the core proteome represents the most prevalent proteins with the high conservancy [40]. Keeping this in mind, the extraction of appropriate candidates for vaccination against ECC using their core genomic content seems logical. As, it provides proteins with high distribution and conservancy among all ECC.

An ideal or promising immunogenic candidate has several criteria. It should be exposed to extracellular space to effectively elicit protective immune responses. Besides, it has to be highly antigenic without any allergenicity. It needs to be highly conserved among widely distributed strains. In addition, an ideal immunogenic candidate should play an important role in the pathogenesis of bacteria and ideally be expressed during bacterial infection [35]. We used the quartile scoring method to subtract our candidates as it provides an option to consider all the above criteria at the same time.

In this study, we introduced nine putative immunogenic proteins as vaccine candidates against ECC, three of which were TonB-dependent proteins. In Gram-negative bacteria, these receptors are involved in the transport and uptake of large substrates such as iron siderophore complexes and vitamin B12 [3]. TonB-dependent receptors are considered excellent candidates for vaccine development due to their critical role in bacterial virulence and vast extracellular exposure. TonB-dependent receptors have been investigated in various Gram-negative pathogens [41]. The TonB-dependent siderophore receptor and the siderophore enterobactin receptor FepA of *Klebsiella* spp. fulfilled all vaccine parameters [42]. In *Acinetobacter baumannii*, two TonB-dependent receptors (BauA and BfnH) showed a partial protective effect on animal models. Aghajani *et al*. indicated that the mortality rate and bacterial load of *A*. *baumannii* in immunized mice was lower compared to that of the control group [43]. YncD of *Salmonella enterica*, an *in vivo*-induced antigen, elicited a significant immune-protection against the lethal wild-type challenge [44]. Furthermore, TbpA of *Neisseria meningitidis* conferred protection against serogroup B [45].

We realized that the TonB-dependent siderophore receptor (WP_058690971.1) has the conserved domain of the ferrichrome outer membrane transporter superfamily belonging to the *Enterobacteriaceae* family. In this study, the *in silico* investigation on ferrichrome porins in different members of *Enterobacteriaceae* demonstrates that they all have the criteria of being vaccine candidates. Despite diverse amino acid compositions of these proteins, our data showed that they are all similar to WP_058690971.1 with regard to physicochemical properties as well as the number of T-cell and conformational B-cell epitopes. The results of immune simulation and molecular docking for these proteins were also close to WP_058690971.1. See S3 Table.

From the other side, proteins belonging to the ferrichrome outer membrane transporter superfamily also have conserved domain of the ligand-gated channel superfamily. This domain was found in four of our candidates including WP_008500981.1, WP_058690971.1, WP_058679571.1 and WP_110108068.1, which had the strongest interaction with TLRs. Their beta-strands are connected with loops on their extracellular side which are required for substrate attachment and transport [46]. It seems that multiple exposed conformational epitopes on the beta-barrel structure of these proteins can interact with the innate immune system effectively. In our previous *in silico* analysis, ligand-gated channel proteins FhuA, BfnH, PapC, DcaP, FatA and IutA were introduced as the best vaccine candidates against *A*. *baumannii*. Therefore, it seems that proteins with beta-barrel structure belonging to ferrichrome outer membrane transporter and ligand-gated channel superfamilies have potential for vaccine development against bacterial pathogens. Accordingly, structure-dependent exploration of vaccine candidates in bacterial genomes could serve as a method for *in silico* vaccine design itself. This method reduces our need for the comprehensive analysis of whole genomes to approach an appropriate vaccine candidate.

Similarly, NlpD conserve domain seem to be an important structure in proteins with vaccine potential. NlpD of WP_025912449.1 has an important role in cell stability. *Yersinia pestis* and *Haemophilus influenzae* lacking NlpD showed less virulence in murine models and failed in growth, respectively [47, 48] Proteins involved in bacterial flagella assembly were among our shortlists. These filamentous structures drive cell locomotion in fluids (swimming process) or on surfaces (swarming), allowing cells to move into favorable environments [49]. In this context, an *in silico* study on *Pseudomonas aeruginosa*, *Morganella morganii* and *Clostridioides difficile* introduced flagellar assembly proteins as valuable vaccine candidates [35, 50, 51]. Therefore, the identification of specific conserved domains in proteins provides essential information regarding their protective potential and possibly estimate their suitability for vaccine design.

## Conclusion

This study introduced nine putative immunogenic candidates against clinical members of ECC using a pan/core-genomic analysis and RV approach. Considering the immune-simulation results, WP_058690971.1, TonB-dependent siderophore receptor, and WP_110108068, YjbH domain-containing protein, were the best immunogenic candidates against ECC with respect to TLRs interactions. However, experimental assays are remaining to determine the efficacy of these candidates. Our investigation introduced ferrichrome porins as ideal vaccine candidates against members of *Enterobacteriaceae*. Proteins belonging to the ferrichrome outer membrane transporter superfamily possess all criteria of suitable vaccine candidates. Therefore, they need to be seriously paid attention to eliminate clinically important *Enterobacteriaceae* by vaccine development. Based on our study, structural-based exploration of vaccine candidates can be considered as a fast and more convenient method for *in silico* vaccine development.

## Supporting information

S1 Table The genetic characteristics and clinical information of twenty *Enterobacter* spp. (DOCX)

S2 Table Physicochemical properties and number of linear B/T-cell and conformational B-cell epitopes for nine putative vaccine candidates against clinical *Enterobacter* spp. (DOCX)

S3 Table Physicochemical properties, number of linear and conformational B/T-cell epitopes, results of immune simulation and TLR binding affinities for eight ferrichrome outer membrane transporters in different members of *Enterobacteriaceae*. (DOCX)

## Abbreviations

CDD: conserved domain database
CDSs: coding sequences
cg-MLST: core genome multi-locus sequence typing
ECC: *Enterobacter cloacae* complex
NJ: neighbor joining
RV: reverse vaccinology
ST: sequence type
UTIs: urinary tract infections
