# Supplementary material for: Subtractive genomic analysis for computational identification of putative immunogenic targets against clinical Enterobacter cloacae complex
Source: PLoS One. 2022 Oct 13;17(10):e0275749. doi: 10.1371/journal.pone.0275749 (PMC9560131; doi:10.1371/journal.pone.0275749)
Supplement: S1 Table — (DOCX) [file pone.0275749.s001.docx]

**S1 Table**. The genetic characteristics and clinical information of twenty Enterobacter spp.

| **Strain name** | **Accession number** | **Genetic characteristics** | **Time of isolation** | **Country of isolation** | **Clinical source** |
| --- | --- | --- | --- | --- | --- |
| *E. asburiae* strain A2563 | AP022628.1 | Total Length: 4,808,368 bp  GC Content: 55.8%  No. of CDSs: 4,527  No. of rRNA: 25  No. of tRNA: 89  Coding Ratio: 88.6% | 2020 | Japan | N/D |
| *E. asburiae* strain Ent 261 | AP024281.1 | Total Length: 4,921,046 bp  GC Content: 55.8%  No. of CDSs: 4,693  No. of rRNA: 25  No. of tRNA: 85  Coding Ratio: 88.5% | 2019 | Japan | Pleural infusion |
| *E. asburiae* strain strain CAV1043 | CP011591.1 | Total Length: 4,753,402 bp  GC Content: 55.7%  No. of CDSs: 4,479  No. of rRNA: 25  No. of tRNA: 85  Coding Ratio: 88.5% | 2008 | USA | N/D |
| *E. cloacae* complex sp. 35734 | CP012162.1 | Total Length: 5,017,289 bp  GC Content: 55.8%  No. of CDSs: 4,733  No. of rRNA: 25  No. of tRNA: 85  Coding Ratio: 89.0% | 2010 | USA | Rectal swab |
| *E. cloacae* strain CBG15936 | CP046116.1 | Total Length: 5,033,927 bp  GC Content (%): 54.7%  No. of CDSs: 4,717  No. of rRNA: 25  No. of tRNA: 86  Coding Ratio: 89.0% | 2017 | China | Sputum |
| *E. cloacae* strain PIMB10EC27 | CP020089.1 | Total Length: 5,272,177 bp  GC Content: 54.5%  No. of CDSs: 4,978  No. of rRNA: 25  No. of tRNA: 87  Coding Ratio: 88.5% | 2010 | Viet Nam | UTI |
| *E. cloacae* strain 109 | CP020525.1 | Total Length: 5,140,725 bp  GC Content: 54.8%  No. of CDSs: 4,885  No. of rRNA: 25  No. of tRNA: 88  Coding Ratio: 88.3% | 2015 | USA | Tracheal aspirate |
| *E. cloacae* strain FDAARGOS 1431 | CP077211.1 | Total Length: 5,316,745 bp  GC Content: 54.8%  No. of CDSs: 5,121  No. of rRNA: 25  No. of tRNA: 91  Coding Ratio: 88.9% | N/D | Germany | N/D |
| *E. roggenkampii* strain FDAARGOS 1430 | CP077407.1 | Total Length: 4,748,459 bp  GC Content: 56.2%  No. of CDSs: 4,361  No. of rRNA: 25  No. of tRNA: 85  Coding Ratio: 89.3% | N/D | Germany | N/D |
| *E. roggenkampii* strain BP10374 | CP038471.1 | Total Length: 4,851,717 bp  GC Content: 56.1%  No. of CDSs: 4,672  No. of rRNA: 25  No. of tRNA: 85  Coding Ratio: 88.5% | 2018 | India | Blood |
| *E. bugandensis* strain 1367 | CP039452.1 | Total Length: 4,750,456 bp  GC Content: 56.0%  No. of CDSs: 4,432  No. of rRNA: 25  No. of tRNA: 85  Coding Ratio: 89.3% | 2011 | Germany | Blood |
| *E. bugandensis* strain 220 | CP039453.1 | Total Length: 4,766,619 bp  GC Content: 56.0%  No. of CDSs: 4,481  No. of rRNA: 25  No. of tRNA: 84  Coding Ratio: 89.2% | 2019 | Germany | Throat swab |
| *E. kobei* strain C16 | CP042578.1 | Total Length: 4,880,257 bp  GC Content: 55.0%  No. of rRNA: 25  No. of tRNA: 88  Coding Ratio: 88.8% | 2009 | Australia | N/D |
| *E. kobei* strain EB_P8_L5_01.19 | CP043511.1 | Total Length: 4,997,888 bp  GC Content: 54.8%  No. of CDSs: 4,772  No. of rRNA: 25  No. of tRNA: 92  Coding Ratio: 89.0% | 2019 | London | N/D |
| *E. kobei* strain DSM 13645 | CP017181.1 | Total Length: 4,880,257 bp  GC Content: 55.0%  No. of CDSs: 4,673  No. of rRNA: 25  No. of tRNA: 88  Coding Ratio: 88.8% | N/D | Japan | blood |
| *E. kobei* strain IB2020 | CP059481.1 | Total Length: 4,801,031 bp  GC Content: 54.7%  No. of CDSs: 4,635  No. of rRNA: 22  No. of tRNA: 85  Coding Ratio: 88.8% | 2019 | Italy | Rectal swab |
| *E. hormaechei* strain AKB48 | CP044335.1 | Total Length: 4,655,363 bp  GC Content: 55.7%  No. of CDSs: 4,337  No. of rRNA: 25  No. of tRNA: 82  Coding Ratio: 89.0% | 2014 | Taiwan | Blood |
| *E. hormaechei* strain 234 | CP021162.1 | Total Length: 4,656,282 bp  GC Content: 55.5%  No. of CDSs: 4,329  No. of rRNA: 25  No. of tRNA: 85  Coding Ratio: 89.3% | 2016 | USA | Wound |
| *E. hormaechei* strain ENCL48880 | CP059419.1 | Total Length: 4,599,702 bp  GC Content: 55.2%  No. of CDSs: 4,280  No. of rRNA: 25  No. of tRNA: 87  Coding Ratio: 88.5% | 2019 | Czech Republic | Sputum |
| *E. cancerogenus* strain FDAARGOS 1428 | CP077290.1 | Total Length: 4,821,734 v  GC Content: 55.7%  No. of CDSs: 4,473  No. of rRNA: 25  No. of tRNA: 87  Coding Ratio: 88.9% | N/D | Germany | N/D |

*N/D = Not Determined
