# Supplementary material for: Subtractive genomic analysis for computational identification of putative immunogenic targets against clinical Enterobacter cloacae complex
Source: PLoS One. 2022 Oct 13;17(10):e0275749. doi: 10.1371/journal.pone.0275749 (PMC9560131; doi:10.1371/journal.pone.0275749)
Supplement: S2 Table — (DOCX) [file pone.0275749.s002.docx]

**S2 Table.** Physicochemical properties and number of linear B/T-cell and conformational B-cell epitopes for nine putative vaccine candidates against clinical Enterobacter spp.

| **Protein Accession**  **Number** | **Protein Length** | **MW**  **(kDa)** | **pI** | **Instability index** | **Hydrophobicity index** | **T-cell epitope**  **ratio** | **No. of T-cell epitopes** | **B-cell epitope**  **ratio** | **No. of B-cell epitopes** | **No. of conformational B-cell** | **Estimated *in vitro* half-life** | **Functional class** |
| --- | --- | --- | --- | --- | --- | --- | --- | --- | --- | --- | --- | --- |
| **WP_008500981.1**  **(TonB-dependent siderophore receptor)** | 749 | 82.87 | 5.42 | 31.4 | -0.638 | 0.11 | 83 | 0.213 | 12 | 4 | >30 hours mammalian  >20 hours yeast  >10 hours *E. coli* | Virulence factor |
| **WP_058690971.1**  **(TonB-dependent siderophore receptor)** | 700 | 77.76 | 5.76 | 36.57 | -0.557 | 0.12 | 88 | 0.107 | 7 | 6 | >30 hours mammalian  >20 hours yeast  >10 hours *E. coli* | Virulence factor |
| **WP_110108068.1**  **(YjbH domain-containing protein)** | 698 | 78.85 | 5.72 | 28.42 | -0.541 | 0.09 | 67 | 0.121 | 8 | 6 | >30 hours mammalian  >20 hours yeast  >10 hours *E. coli* | Cellular process |
| **WP_058679571.1**  **(TonB-dependent vitamin B12 receptor BtuB)** | 617 | 68.55 | 5.71 | 28.81 | -0.538 | 0.1 | 67 | 0.106 | 5 | 8 | >30 hours mammalian  >20 hours yeast  >10 hours *E. coli* | Cellular process |
| **WP_088207510.1**  **(Flagellar hook-associated protein FlgK)** | 547 | 57.63 | 4.66 | 28.1 | -0.269 | 0.15 | 87 | 0.202 | 10 | 5 | >30 hours mammalian  >20 hours yeast  >10 hours *E. coli* | Cellular process |
| **WP_033145204.1**  **(Flagellar hook protein FlgE)** | 416 | 43.42 | 4.8 | 14.73 | -0.253 | 0.12 | 54 | 0.161 | 6 | 5 | >30 hours mammalian  >20 hours yeast  >10 hours *E. coli* | Virulence factor |
| **WP_058679632.1**  **(Flagellar hook length control protein FliK)** | 411 | 41.77 | 4.92 | 44.88 | -0.236 | 0.09 | 39 | 0.515 | 9 | 3 | >30 hours mammalian  >20 hours yeast  >10 hours *E. coli* | Cellular process |
| **WP_039266612.1**  **(Pore coat U domain-containing protein)** | 321 | 33.84 | 7.45 | 20.91 | 0.088 | 0.13 | 42 | 0.19 | 5 | 3 | >30 hours mammalian  >20 hours yeast  >10 hours *E. coli* | Cellular process |
| **WP_025912449.1**  **(Peptidoglycan DD-metalloendopeptidase family protein)** | 247 | 26.13 | 9.89 | 22.14 | -0.278 | 0.11 | 28 | 0.21 | 2 | 2 | >30 hours mammalian  >20 hours yeast  >10 hours *E. coli* | Virulence factor |

*MW: Molecular weight
