# Supplementary material for: Subtractive genomic analysis for computational identification of putative immunogenic targets against clinical Enterobacter cloacae complex
Source: PLoS One. 2022 Oct 13;17(10):e0275749. doi: 10.1371/journal.pone.0275749 (PMC9560131; doi:10.1371/journal.pone.0275749)
Supplement: S3 Table — (DOCX) [file pone.0275749.s003.docx]

**S3 Table.** Physicochemical properties, number of linear and conformational B/T-cell epitopes, results of immune simulation and TLR binding affinities for eight ferrichrome outer membrane transporters in different members of Enterobacteriaceae.

| **Protein Accession** | **TM** | **Organism** | **Adhesin Probability** | **No of AA** | **MW** | **pI** | **Instability**  **index** | **HI** | **No of T-cell epitopes** | **T-cell**  **epitope ratio** | **No of linear B-cell epitopes** | **B-cell epitope Ratio** | **No. of conformational B-cell epitopes** | **IFN-γ**  **(ng/ml)** | **Th1**  **(cells/mm^3^)** | **IgM**  **(cells/mm^3^)** | **IgG1**  **(cells/mm^3^)** | **TLR-1**  **(kcal/mol)** | **TLR-2**  **(kcal/mol)** | **TLR-4**  **(kcal/mol)** |
| --- | --- | --- | --- | --- | --- | --- | --- | --- | --- | --- | --- | --- | --- | --- | --- | --- | --- | --- | --- | --- |
| WP_012134008.1 | 0 | *C. koseri* | 0.79 | 749 | 82.53 | 5.38 | 29.87 | -0.494 | 78 | 0.1 | 8 | 0.12 | 10 | 370000 | 39000 | 1 | 6 | -61.21 | -46.637 | -57.552 |
| WP_000124388.1 | 0 | *E. coli* | 0.69 | 731 | 81.16 | 5.6 | 38.01 | -0.501 | 76 | 0.1 | 7 | 0.1 | 7 | 380000 | 39000 | 1 | 10 | -69.594 | -55.115 | -69.594 |
| WP_000124438.1 | 0 | *E. coli* | 0.72 | 747 | 82.18 | 5.47 | 30.04 | -0.481 | 76 | 0.1 | 8 | 0.11 | 7 | 390000 | 39000 | 1 | 11 | -49.351 | -59.082 | -55.683 |
| WP_004178624.1 | 0 | *K. pneumoniae* | 0.73 | 735 | 81.38 | 5.66 | 34.98 | -0.521 | 66 | 0.08 | 6 | 0.08 | 7 | 390000 | 39000 | 1 | 11 | -56.16 | -52.449 | 37.519 |
| WP_012542816.1 | 0 | *K. pneumoniae* | 0.75 | 735 | 81.3 | 5.43 | 35.03 | -0.503 | 70 | 0.09 | 7 | 0.08 | 6 | 390000 | 39000 | 0 | 8 | -59.123 | -55.684 | -69.338 |
| WP_000113211.1 | 0 | *S.enterica* | 0.71 | 747 | 82.29 | 5.35 | 28.59 | -0.439 | 82 | 0.1 | 9 | 0.1 | 5 | 380000 | 39000 | 1 | 9 | -59.123 | -51.406 | -57.074 |
| WP_011587185.1 | 0 | *S.flexneri* | 0.75 | 721 | 79.5 | 5.13 | 29.27 | -0.502 | 69 | 0.09 | 7 | 0.095 | 8 | 370000 | 39000 | 0 | 11 | -76.856 | -57.307 | -62.277 |

*HI: Hydrophobicity index; TM: Transmembrane helices.
